# The boom and bust cycle of biobanking – thinking through the life cycle of biobanks

**DOI:** 10.3325/cmj.2013.54.501

**Published:** 2013-10

**Authors:** Aaro Tupasela, Neil Stephens

**Affiliations:** 1 Department of Social Research, University of Helsinki, Helsinki, Finland; 2 CESAGEN, Cardiff University, Cardiff, UK

The collection and use of tissue samples is by no means a new phenomenon (1,2). As an object of political interest and action, the international preoccupation and focus on biobanking has grown significantly in recent years. What has become more apparent is the quest to develop a more robust and systematized system for the collections, storage, and distributions of tissue samples and the data that can be derived from them. From a policy perspective it is clear that biobanking has attracted a great deal of public funding in order to set up international networks of exchange between various actors. At the same time, however, the international boom associated with biobanks raises a number of important questions as to the development and sustainability of the field in the long run.

As part of the “Bio-objects” research network action supported by the European Cooperation in Science and Technology (COST) program, the Working Group on Generative Relations is interested in the different ways in which various processes of bio-objectification become productive in society. In this sense, the role of biobanks as a nexus of productive capacity located between the populations and biomedical research is seen to be politically and scientifically crucial. During the past years, the interest to set up new biobanks in order to become a part of the development of international networks for exchange and research has become evident (3), where national innovation and research policies have played an important part in supporting such developments (4). There has also been a surge in the development of national systems for the collection and exchange of tissue samples and related material around the word. These processes include questions as to how to set up collection and storage facilities, what quality standards to use, as well as what types of pricing models should be implemented in order to keep the collection, storage, and distribution process sustainable.

With the surge in setting up new biobanks it is important to ask to what degree have those who are entrusted with managing these collections considered the long-term sustainability of such collections when there is likely to be fierce competition among actors to collect and maintain their own collections. How many biobanks prepare for situations where the possibility of maintaining a collection becomes too expensive? What types of safeguards are in place to prepare for financial hardships, transfer of material and information to new actors, or the possibility of shutting down of biobanks? To what degree do national funding organizations consider overlap?

## Long-term sustainability

The long-term policy goal associated with supporting biobanking activities and the development of biobanking infrastructures throughout Europe relates to the development of new innovations and business opportunities based on biobanking research. Diagnostics, pharmaceutical development, and therapeutics are only some of the areas to benefit from biobanking. It is, however, unclear to what degree national biobanking initiatives overlap and reproduce efforts, which are being undertaken in other countries and even within the same country. Although this can be argued to create a competitive environment, it also represents a waste of public funding and a situation in which the fate of many biobanks is tenuous at best. In many cases the long-term possibilities of running biobanks will depend on the continued support of public funding organizations and institutions. It is unclear, however, to what extent various funding organizations and institutions, such as hospitals, will be willing to support such activities if there are few concrete returns.

The productivity of biobanks is another major problem facing the field in general. Although biobanks have existed for decades in various forms, it is only recently, with the political attention that they have gained, that the question of measuring productivity has come up. Increased public funding is followed by increased scrutiny over impact on society, still it is unclear how the productivity of biobanks should and could be measured. Should there be some form of Biobank Impact Factor (BIF) (5) or Bioresource research Impact Factor (BRIF) (gen2phen) that could be compared across borders, and in what ways would one measure different types of biobanks, such as stem cell banks, cord blood banks, epidemiological biobanks, and diagnostic biobanks?

Due to the infancy of the field in terms of translating research into products, the benefits of biobanking are very difficult to operationalize in economic terms. DeCode Genetics, for example, has been one of the leading companies in publishing high quality scientific articles, yet its track record in securing income from innovations is still very modest in relation to the investment that has gone into its activities. During times of economic recession and hardship, it is natural that public organizations, such as hospitals will pay close attention to the ways in which tax-payer money is being spent and the impact that that money has on public health and medical practices.

## When biobanks fail

Inevitably, with such a large number of biobanks being established, some will subsequently close, or be substantially reconfigured. This brings with it a particular set of ethical challenges that practitioners and social scientists must contend with (6), that should make a core contribution to any “sociology of biobanking” (7).

Cadigan et al (8) report examples of biobank managers who struggle with the discrepancy of hoping their bank will exist permanently while knowing their funding is limited. People in management positions are right to be aware of potential closure. Examples where this has already occurred within the private sector include SeraCare Life Sciences that sold its biobank in 2013 to refocus upon producing in vitro diagnostics products (9,10), Ardais that collapsed in 2005, selling on its biosamples to Cytomyx Holdings and its bespoke software to Gulfstream (11), and the Swedish project UmanGenomics that was mothballed in 2003 (12). Closures are not confined to the private sector, with public sector examples including the Massachusetts Stem Cell Bank that closed in 2012 (13), the Singapore Biobank and the US Armed Forces Institute of Pathology that closed in 2011 (14,15), and the UK Human Tissue Bank that closed in late 2009 (16). When these banks close the processes of bio-objectification they engage in also breakdown as the value attached to the tissue shifts in the absence of institutional structure, standards, and best practice.

Neil Stephens, co-author of this paper, has conducted empirical work observing the closure of a biobank in real time. The biobank – which must remain anonymous due to the terms of the research – was established to both collect high quality disease specific human tissue and to operate as a focal point for developing national standards for best practice in the field. Initially well funded through governmental and charity sources, the biobank progressed well in negotiating access to tissue with hospitals. However, over time rigidities within these hospitals led to less tissue being delivered into the biobank than initially anticipated and, against a backdrop of increasing financial tightening, the funders decided to cease their support for biobanking activity. Like SeraCare Life Sciences, Ardais, and the US Armed Forces Institute of Pathology, the biobank was then fragmented as parts of its tissue holding, computer software, and human resources were moved and reused in new biobanking contexts. It is clear that the boom and bust cycle of biobanking can bring institutions to an end. But it is also possible that after closure elements of a biobanking infrastructure can find a new use elsewhere.

As discreet entities biobanks perform a particular type of bio-objectification that brings together the sources of material and information with users and refiners of that material. At the same time, however, biobanks are supposed to exemplify one of the core suppositions of ideologies of the knowledge-based bio-economy where increasingly biological resources can be exploited and developed to the benefit of society through the commercialization and commodification of new types of products that become socially and economically productive. It is, however, unclear at this point to which degree bio-banks will be able to generate new knowledge on diseases that can be exploited and utilized in the health care sector and products and services.
